# The Open Workshop Method in Nature in The Eco Art Therapy Project

**DOI:** 10.1192/j.eurpsy.2026.11458

**Published:** 2026-07-31

**Authors:** E. Chirila

**Affiliations:** Filiala Cluj, Uniunea Artistilor Plastici Din Romania, Cluj-Napoca , Romania

## Abstract

**Introduction:**

One of the most obvious developments in the field of art therapy over the last decade has been the return to the workshop and the development of a variety of workshop-based approaches (such as those described by Pat Allen, Carl Rogers, and Shaun McNiff ), approaches that incorporate the ideas of Rudolf Arheim in “Art and Visual Perception” and especially in “Gestalt Psychology and Artistic Form,” which in turn are related to the informational aesthetics of Abraham Moles and Max Bense, for whom information is a putting into form.

Ecological/existential factors are analyzed, the relationship with the surrounding community is established: we benefit from a more complete

circulation and ecology of expressive energy because we can see how the mechanisms of change allow for the holistic transformation of the beneficiary.

**Objectives:**

Through the “OPEN WORKSHOP IN NATURE” method, the artist is a co-creator with the beneficiaries, approaching the activities on multiple levels of adaptability. The holistic approach to well-being aims to allow beneficiaries, who are in fragmented states, to achieve integrity, unity, harmony and balance, taking into account all the mental, emotional, physical and spiritual dimensions of human nature.

**Methods:**

One of the basic ideas of creative-visual is that many of the scientific interdisciplinary approaches can be learned and experimented using the creative process. Following the medical and psychological diagnosis, the artist-art designer-therapist establishes a therapeutic protocol, which can complement the diagnosis (the “Forestier’s Sheet” is known) J. Rodriguez and G. Troll Ecological / existential factors are analyzed, the relationship with the surrounding community is facilitated: we benefit from a more complete circulation and ecology of expressive energy because we can see how the mechanisms of change allow the holistic transformation of the beneficiary.

**Results:**

The beneficiary is assisted in his/her development regarding the concepts of self, environment and its functioning.

The formative results are intended to endow the person with new valences of plenary living and during the active period – regardless of the chosen career, but also later, throughout life.

**Image 1:**
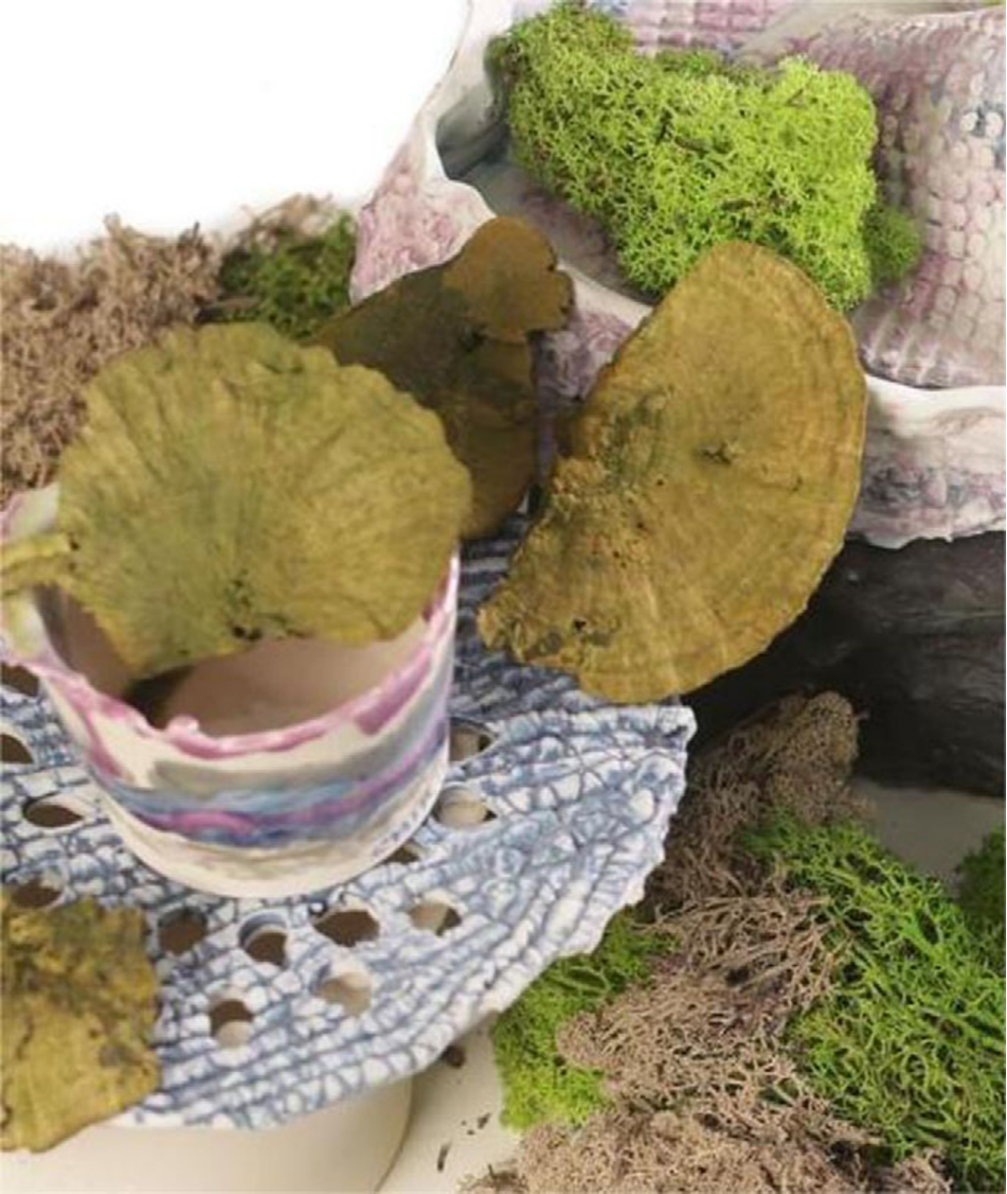

**Image 2:**
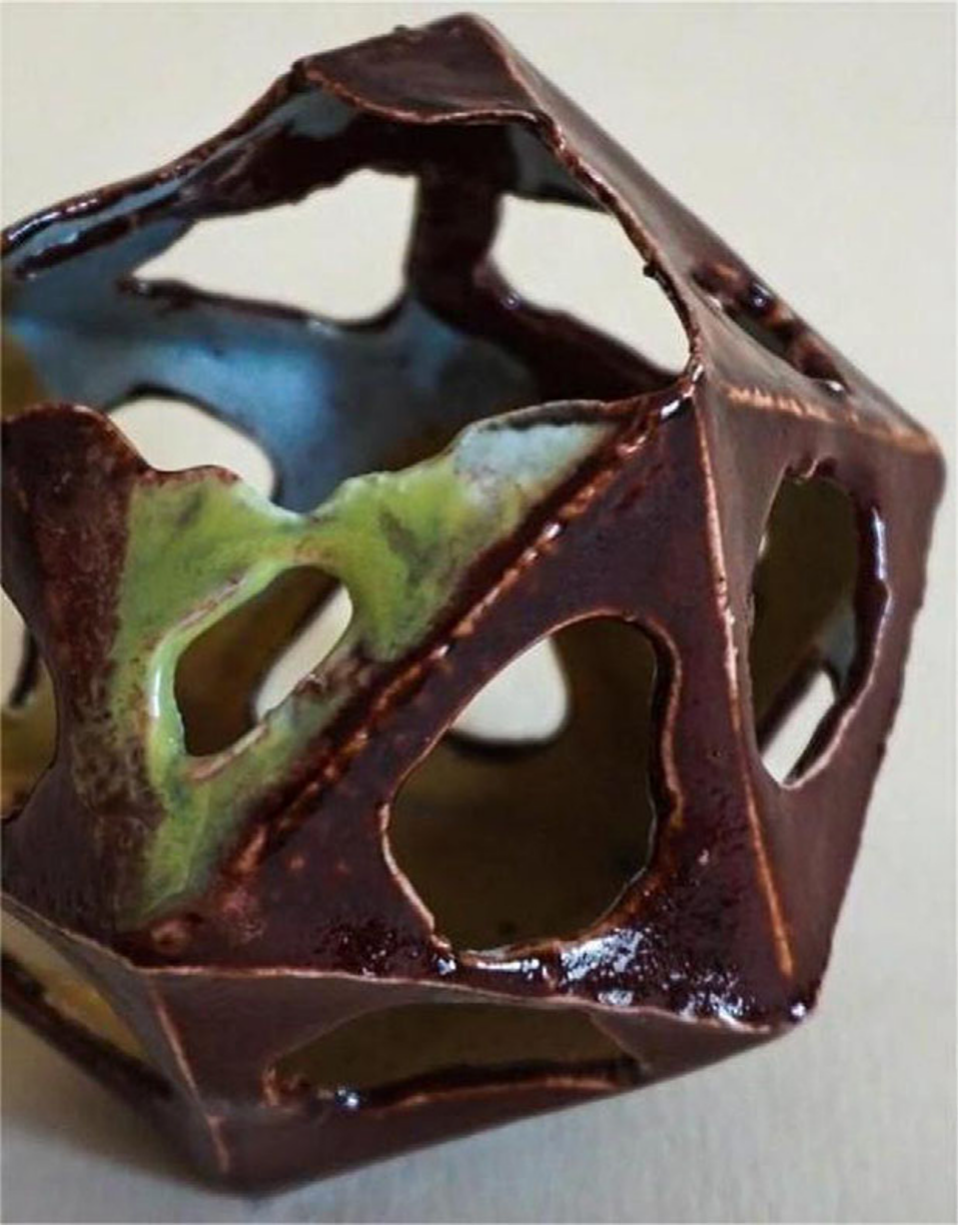

**Image 3:**
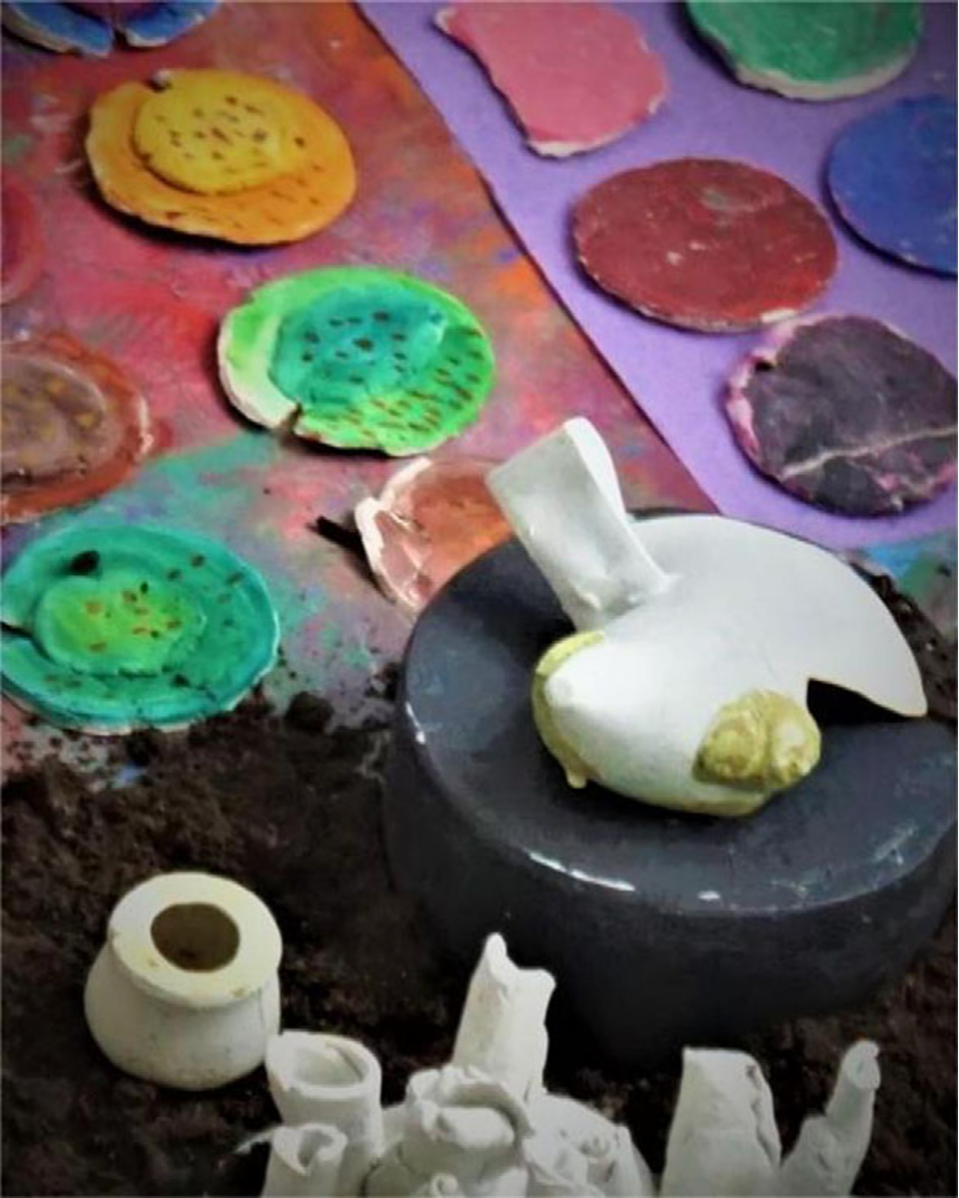

**Conclusions:**

When the primary perception of form in nature (which constitutes the simplest form of understanding) triggers a process that continues within a phenomenon analyzed individually or by a group, then the apparent phenomenon disappears, coagulates (merges, interpenetrates), forming, giving rise to, a model form, which is the bearer of the most varied and profound information about nature. The discovery, the formulation of the idea, often lies at the roots of science and art. The development of Western landscape art can be seen by future art historians as the sign of an emerging eco-consciousness and the avatar of future eco-cultural practices.

**Disclosure of Interest:**

None Declared

